# Exceptional Photocatalytic Activities of rGO Modified (B,N) Co‐Doped WO_3_, Coupled with CdSe QDs for One Photon Z‐Scheme System: A Joint Experimental and DFT Study

**DOI:** 10.1002/advs.202102530

**Published:** 2021-12-03

**Authors:** Fazal Raziq, Amil Aligayev, Huahai Shen, Sharafat Ali, Rahim Shah, Sajjad Ali, Syedul H. Bakhtiar, Asad Ali, Naghat Zarshad, Amir Zada, Xiang Xia, Xiaotao Zu, Muslim Khan, Xiaoqiang Wu, Qingquan Kong, Chunming Liu, Liang Qiao

**Affiliations:** ^1^ Yangtze Delta Region Institute (Huzhou) University of Electronic Science and Technology of China Huzhou 313001 P. R. China; ^2^ School of Physics University of Electronic Science and Technology of China Chengdu 610054 P. R. China; ^3^ Institute of Nuclear Physics and Chemistry Chinese Academy of Engineering Physics Mianyang 621900 P. R. China; ^4^ Department of Physics Southern University of Science and Technology Shenzhen 518055 P. R. China; ^5^ The State Key Laboratory of Advanced Technology for Materials Synthesis and Processing Wuhan University of Technology Wuhan 430070 P. R. China; ^6^ Department of Chemistry Abdul Wali Khan University Mardan KPK 23200 Pakistan; ^7^ Department of Chemistry Kohat University of Science and Technology Kohat KPK 26000 Pakistan; ^8^ School of Mechanical Engineering Chengdu University Chengdu 610106 P. R. China

**Keywords:** CdSe quantum dots, expending visible‐light response, one‐photon Z‐scheme, overall‐water splitting, surface modifications, WO_3_

## Abstract

Artificial Z‐scheme, a tandem structure with two‐step excitation process, has gained significant attention in energy production and environmental remediation. By effectively connecting and matching the band‐gaps of two different photosystems, it is significant to utilize more photons for excellent photoactivity. Herein, a novel one‐photon (same energy‐two‐photon) Z‐scheme system is constructed between rGO modified boron‐nitrogen co‐doped‐WO_3_, and coupled CdSe quantum dots‐(QDs). The coctalyst‐0.5%Rh*_x_* Cr_2_O_3_(0.5RCr) modified amount‐optimized sample 6%CdSe/1%rGO3%BN‐WO_3_ revealed an unprecedented visible‐light driven overall‐water‐splitting to produce ≈51 µmol h^−1^ g^−1^ H_2_ and 25.5 µmol h^−1^ g^−1^ O_2_, and it remained unchanged for 5 runs in 30 h. This superior performance is ascribed to the one‐photon Z‐scheme, which simultaneously stimulates a two photocatalysts system, and enhanced charge separation as revealed by various spectroscopy techniques. The density‐functional theory is further utilized to understand the origin of this performance enhancement. This work provides a feasible strategy for constructing an efficient one‐photon Z‐scheme for practical applications.

## Graphical abstract

The design of a novel Z‐scheme system based on a band gap adjusted visible‐light responsive 0.5RCr/6CdSe/1rGO/3BN‐WO3 nanocomposite is successfully constructed via hydrothermal method. This work demonstrates a promising approach to synthesize nanophotocatalysts based on WO3 for visible‐light driven solar energy application.

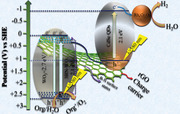

## 1 Introduction

The increasing energy demand and environmental security due to climate change are the biggest challenges that mankind facing today. It is irresistible to develop sustainable, clean, and environmentally friendly energy resources.^[^ ^1^, ^2^, ^3^, ^4^, ^5^, ^6^ ^]^ Solar energy is rich in terms of its availability to satisfy present and future world‐wide energy needs.^[^ ^7^, ^8^, ^9^ ^]^ Nevertheless, it is quite indispensable to change solar energy into other forms that are stress‐free to stock, transport, and utilize. Amongst numerous approaches, valuable H_2_ gas evolved by solar driven water‐splitting is genuinely auspicious.^[^ ^10^, ^11^, ^12^, ^13^ ^]^ Since the phenomena was discovered for water splitting on a single crystal semiconductor (TiO_2_) in 1972,^[^ ^14^ ^]^ afterward various semiconductor photocatalysts have been widely investigated.^[^ ^15^, ^16^ ^]^ However, it is extremely challenging for a single material to catalyze practical water splitting with high efficiency, because of the requirement of two criteria: 1) broad range solar light absorption; and 2) durable thermodynamic torrential force for photolytic applications.^[^ ^17^, ^18^ ^]^

In recent years, the use of two semiconductor systems with different band gaps and adjustable donor/acceptor pairs (so‐called shuttle redox mediator) are gaining more research attention due to its enhanced activity. Therefore, the photocatalytic water splitting via two‐step photoexcitation has become a promising strategy for artificial photosynthesis. This system is in analogy to the natural photosynthesis of green flora and is called the “Z‐scheme”.^[^ ^19^, ^20^ ^]^ The Z‐scheme system contains two individual photocatalysts, that is, BiVO_4_/RuSrTiO_3_^[^ ^21^ ^]^ Rh‐Pt‐WO_3_/Pt‐SrTiO_3_^[^ ^22^ ^]^ (Cr, Ta) and Pt‐SrTiO_3_^[^ ^23^ ^]^ Rh‐SrTiO_3_/BiVO_4_.^[^ ^23^ ^]^ The theoretical Z‐scheme proves to absorb more light than the single photocatalyst. However, the main drawback of Z‐scheme is the band gap mismatch of the two different semiconductors, mostly one component has narrow and another component has wide band gap. Therefore, it is highly imperative to adjust the band gaps of two different systems in the artificial Z‐scheme in order to improve the synergistic effects of wide‐range solar‐spectrum utilization and thermodynamic pouring force for photocatalytic reactions.^[^ ^24^, ^25^ ^]^

Extensive research based on the metal‐oxide semiconductor nanoparticles, such as zinc oxide (ZnO),^[^ ^26^ ^]^ tin oxide (SnO_2_),^[^ ^27^ ^]^ indium oxide (In_2_O_3_),^[^ ^28^ ^]^ tungsten oxide (WO_3_),^[^ ^29^ ^]^ molybdenum oxide (MoO_3_),^[^ ^30^ ^]^ and vanadium oxide (V_2_O_5_),^[^ ^31^ ^]^ have been conducted to promote the photocatalytic conversion efficiency. Among these oxide materials, WO_3_, an n‐type semiconductor, is deliberated as a vanguard material for photocatalysis.^[^ ^32^, ^33^ ^]^ It has several distinguished advantages, such as the relatively narrow energy gap (2.7 eV) than other oxides, high oxidation potential (+3.1–3.2 VNHE) of the valence band (VB), cost‐effective, environmentally friendliness, optical chemical performance, good photo stability, and biocompatibility.^[^ ^34^, ^35^, ^36^ ^]^ Nevertheless, its applications are restricted since it can't be employed for H_2_ production owing to its lower conduction band (CB) potential, insufficient absorption of light from solar spectrum, and high rate of excited charge recombination. However, these limitations could be overcome by microstructure and composition modifications, such as semiconductor coupling, noble metal deposition, and elemental doping et al. Among them, doping is an effective strategy for narrowing the band gap and improving the photocatalytic activities. Traditionally, doping of WO_3_ can be realized by substituting W and O atoms, respectively, with transition metal cations (Cr, Co, Gd, Fe)^[^ ^37^, ^38^, ^39^, ^40^ ^]^ and non‐metal anions (B, N, S, C, P)^[^ ^41^, ^42^, ^43^, ^44^ ^]^ in order to tune CB and valence band (VB) positions, modulating optical absorption, charge separation, hydrophilicity, and defect density. Of these elements, anion B or N doping has shown promising results in changing the CB and VB, respectively, and boosting the photocatalytic activities. N is the most widely studied dopant that has an atomic radius (155 pm) similar to oxygen (152 pm) while B with low electronegativity can generate spatially localized resonant states, pairs, and clusters in band gap and thus create a large energy shift for CB of WO_3_.^[^ ^45^ ^]^ In addition, non‐metal elements are relatively less toxic which represents an eco‐friend strategy to enhance the optical and electrical properties. However, the photocatalytic efficiency of the modified WO_3_ with single dopant is still limited by the slow charge transfer and the speedy recombination of a photo‐generated charge carrier pair. In this respect, various efforts have been used to engineer WO_3_ in order to enhance charge transfer, reduce power, and effectively reduce carrier recombination. In this regard, the emergent 2D material graphene (rGO) is a promising candidate owing to its high carrier mobility, efficient electron (e^−^) collection, and transportation properties. In addition, towards practical overall water splitting applications, co‐catalyst can further improve the redox reactions of one photon‐Z‐scheme system.

In this work, we co‐doped WO_3_ with B and N anions to tune its band gap. The photocatalyst was further modified with rGO as a charge carrier's transporter, and coupled with CdSe quantum dots (QDs) to develop an efficient one‐photon Z‐scheme system. Further, the optimized sample 6CdSe/1rGO/3BN‐WO_3_ was loaded with 0.5%Rh*_x_* Cr_2_O_3_ (0.5RCr) as a co‐catalyst by photodeposition method. The obtained results show that 0.5RCr modified 6CdSe/1rGO/3BN‐WO_3_ has excellent photocatalytic activity for overall water splitting as well as for pollutant degradation. Both components of this novel Z‐scheme system were excited at the same energy photon. This configuration increased the lifetime of photo‐generated charges and efficiency of photocatalysis beyond 580 nm in the visible‐light range, which is ascribed to the synergistic effect of CdSe QDs coupling and BN co‐doping. Density functional theory (DFT) calculation demonstrates the role of dopants to create allowed surface states in the band gap. Surface states possess small ionization energies and when the doping charge density is high, the dopant generates new states below the conduction and above the valence band edges to reduce the band gap. Based on these experimental and theoretical investigations, we believed that the newly prepared RCr/CdSe/rGO/BN‐WO_3_‐based nanocomposite will have broad applications for solar fuels evolution and environmental applications.

## 2 Computational Details

The current study has been carried out through the spin‐polarized DFT calculations using generalized gradient approximation (GGA) in the form of Perdew‐Burke‐Ernzerhof (PBE)^[^ ^46^ ^]^ for the exchange‐correlation potentials, as implemented in the Vienna ab initio simulation package (VASP).^[^ ^47^, ^48^, ^49^ ^]^ A dense mesh of 4 × 4 × 4 *k*‐points and 450 eV energy cut‐off has been used to optimize structures of pristine and modified WO_3_. The projector augmented wave (PAW) formalism^[^ ^50^, ^51^ ^]^ was used to model the ion–electron interactions, and Grimme's dispersion correction (DFT‐D3) was applied to account for van der Waals interactions.^[^ ^52^ ^]^ The total energy was converged to an accuracy of 1 × 10^−5^ eV to obtain accurate forces, and a force tolerance of 0.02 eV Å^−1^ was applied in the structure optimization. The HSE06 functional was used to calculate the band gap, and the *k*‐point mesh was set to 6 × 6 × 6. The unit cell of WO_3_ structure contains 8 W and 24 O‐atoms with orthorhombic W (Pmnb space group) contained in a partial oxygen octahedron structure.

## 3 Results and Discussion

### 3.1 DFT Calculations

**Figure 1:**
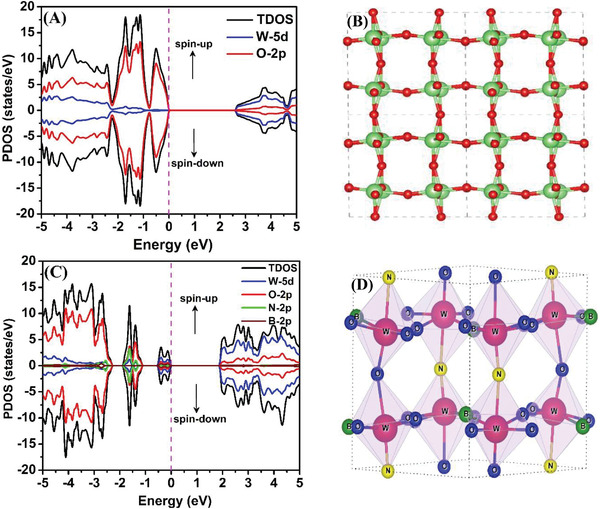
The spin‐polarized total density of states (TDOS) and partial density of states (PDOS) plotted together with corresponding optimized structures of (A,B) bare WO3 and (C,D) nitrogen and boron co‐doped WO3, respectively. The spin‐up (↑) and spin‐down (↓) states are indicated by positive and negative values, respectively. The Fermi level (EF) is set to zero.

We first examined the structure and electronic properties of pure WO_3_ and BN co‐doped WO_3_ using PBE and HSE functionals. A 2 × 2 × 2 supercell comprising 256 atoms in orthorhombic phase is shown in **Figure** **1**B,D. In fact, in the orthorhombic phase, W represents a perovskite‐like geometric structure. Compared to the triclinic crystalline structure, the long‐short “W—O” bond length in the orthorhombic WO_3_ is oriented only in the *y* and *z* directions, because of the W‐atom drifting from its equilibrium location. We found that the HSE and PBE hybrid functional yield lattice constants and symmetry volumes that match the experimental data are much better than the standard DFT‐generalized gradient approximation (GGA) functional. Most importantly, the introduction of a portion of exact HF exchange energy in the hybrid functional always gives a systematically precise band gap description which is underestimated by conventional DFT methods. To determine the most stable structure of (B,N) co‐doped WO_3_ for the electronic calculations, we considered all possible combinations of O substitution for B and N and choose the one with lowest total energy for further calculations. The HSE06 functional is used to investigate and compare the electronic band gap of both pristine and B and N co‐doped WO_3_ as shown in Figure 1. The spin‐polarized total density of state (TDOS) and partial density of state (PDOS) of W‐d, O‐P, N‐P, and B‐P orbitals are calculated and plotted together with corresponding structures, as shown in Figure 1. The electronic density of states (DOS) analysis revealed that both WO_3_ and 3BN‐WO_3_ are semiconductors with relatively narrow band gaps of 2.7 and 2.1 eV, respectively, as shown in Figures 1A and 1C. Furthermore, it reveals that the (B,N) co‐doping in WO_3_ produces delocalized electrons, leading to a high band gap reduction which makes WO_3_ as a highly efficient catalytic material. The d‐orbital of W atom hybridizes with p‐orbitals of the surrounding O, N, and B atoms resulting in lowering the band gap of 3BN‐WO_3_ materials. An intriguing outcome of hybrid‐functional examination reveals that the band gap value is muscularly dependent on the structure.

### 3.2 Structure Characterization and Chemical Composition

The crystal structure of the pure WO_3_, 3BN‐WO_3_, 1rGO/3BN‐WO_3_, 6CdSe/1rGO/3BN‐WO_3_, and co‐catalyst modified 0.5RCr/6CdSe/1rGO/3BN‐WO_3_ photocatalysts were investigated by X‐ray powder diffraction (XRD) in the range of 10° to 90° as given in Figure S1A, Supporting Information. The bare WO_3_ exhibits a monoclinic crystal phase with well resolved triplet diffraction peaks at 2*θ* = 23.22°, 23.72°, and 24.46° attributed to (002), (020), and (200) planes, respectively, as confirmed by X‐ray diffraction (JCPDS card No. 72–0677). Furthermore, the plain W represents the space group P2_1_/c, with lattice parameters a = 7.3271 Å, b = 7.5644 Å, c = 7.7274 Å, and beta = 90.488°. Interestingly, the WO_3_ sample shows no changes in the crystal phase or interlayer distance after BN doping, and no additional peak or phase related to the presence of BN compound was observed. The potential reason is that oxygen and nitrogen have the similar atomic radii suggesting that the incorporation of BN pairs into a WO_3_ six‐membered ring is successful. Because of the low content, the rGO diffraction peaks are absent in the 1rGO/3BN‐WO_3_ composites. The yCdSe/1rGO/3BN‐WO_3_ nanocomposite shows no extra peak after coupling with CdSe QDs, indicating that the CdSe QD composition is very low and the composite maintains the original microstructure. Therefore, the well‐defined XRD peaks show that the as‐prepared nanocomposites have high crystallinity with no impurity phase.

**Figure 2:**
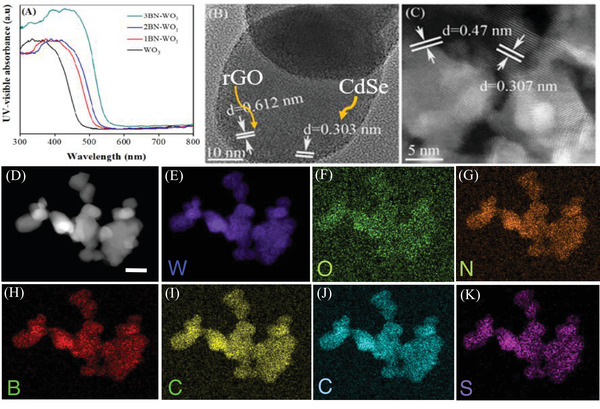
The UV–visible absorption spectra of WO_3_, 1BN‐WO_3_, 2BN‐WO_3_, and 3BN‐WO_3_ (A), HR‐TEM images of 6CdSe/1rGO/3BN‐WO_3_ (B) and (C), and elementary mapping of the optimized‐sample 6CdSe/1rGO/3BN‐WO_3_ (D–L).

The UV–Vis absorbance spectra were measured to explore the optical properties and electronic band gaps of the WO_3_, 1BN‐WO_3_, 2BN‐WO_3_, and 3BN‐WO_3_ photocatalysts as shown in **Figure** **2**A while the UV–Vis absorbance spectra for the samples after coupling with CdSe QDs and photo‐deposited RCr are shown in Figure S1B, Supporting Information. The absorption spectra show significant increase in intensity above 470 nm after (B,N) co‐doping, with the threshold shifting toward longer wavelengths. It suggests that the band gaps of the pristine WO_3_ have been modified. Furthermore, when coupled with CdSe QDs, the visible light response of BN‐WO_3_ is dramatically extended up to 580 nm. The Kubelka–Munk method was used to estimate the band gap energies of all the samples, and the determined band gaps for WO_3_, 3BN‐WO_3_, and CdSe QDs are 2.7, 2.1, and 2.1 eV, respectively. For comparison, the electronic properties and band gaps of WO_3_ and 3BN‐WO_3_ were calculated by using DFT as discussed earlier in Figure 1. The obtained band gaps for the pristine WO_3_ and 3BN‐WO_3_ are 2.8 and 2.1 eV, respectively, which are consistent with the experimental values. The presence of (B,N) dopants in WO_3_ results in a stable and suitable energy state, demonstrating the thermodynamic stability of BN‐WO_3_. The details of the electronic band structure of the nanocomposite system will be extensively discussed in Figure 6.

Further structural exploration of the nanocomposite was revealed by transmission electron microscope (TEM)/HRTEM investigations. The TEM images demonstrate that no change in morphology is observed after the introduction of BN as shown in Figure S2, Supporting Information. The distribution of rGO and CdSe QDs on the surface of 3BN‐WO_3_ can be clearly observed from TEM images. The lattice fringes of CdSe QDs, rGO, and BN doped WO_3_ are visible in the HRTEM in Figure 2B,C. These figures demonstrate that CdSe QDs in the 3–5 nm size range are inhomogeneously dispersed on the surface of nanocomposite. From HRTEM images, it is also confirmed that rGO and CdSe QDs are chemically bonded with 3BN‐WO_3_. Moreover, elemental mapping analysis was performed to investigate elemental composition and homogeneous distribution of the constituent elements. Figure 2D–L demonstrates that the composite contains regularly distributed Cd, Se, C, B, N, W, and O elements. Furthermore, as shown in Figure S3, Supporting Information, the energy‐dispersive X‐ray spectroscopy (EDX) results show that all of the elements are present in appropriate concentrations with no additional impurity peaks.

**Figure 3:**
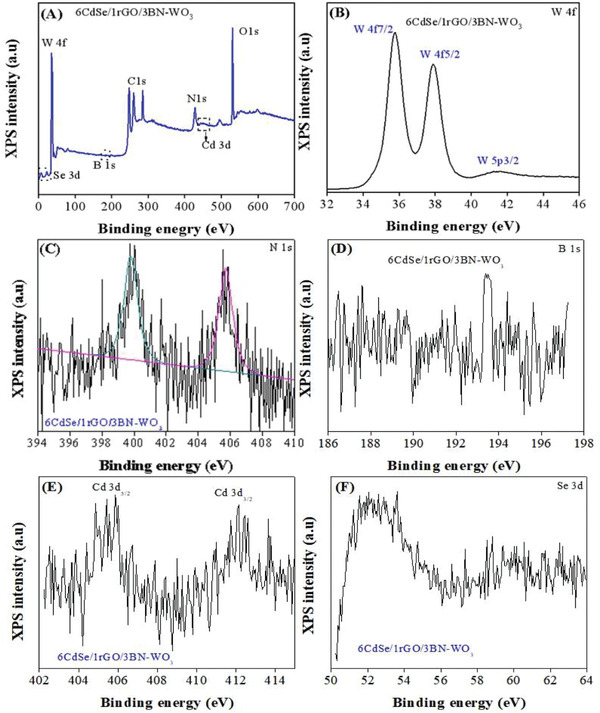
The XPS survey spectra of 6CdSe/1rGO/3BN‐WO_3_ (A), the high‐resolution spectra of W 4f (B), N1s (C), B1s (D), Cd 3d (E), and Se 3d of 6CdSe/1rGO/3BN‐WO_3_ (F).

X‐ray photoelectron spectroscopy (XPS) was used to examine the chemical valence and bonding state of elements in 6CdSe/1rGO/3BN‐WO_3_ sample. The spectra of the optimized nanocomposite and peaks for Cd, Se, C, B, N, W, and O are shown in **Figure** **3** and Figure S4, Supporting Information. The XPS survey spectrum demonstrates that all the constituent elements are present and no additional peaks are observed in the nanocomposites (Figure 3A). The W 4f spectrum is identified at 35.8, 37.9, and 41.6 eV binding energies and, respectively, coordinated with W4f_7/2_, W4f_5/2_, and W5p_3/2_ indicating a +6 oxidation state of tungsten as shown in Figure 3B, which is consistent with previous reports.^[^ ^53^ ^]^ The O1s spectrum can be deconvoluted into two main peaks at 530.8 and 531.7 eV as shown in Figure S4A, Supporting Information. The 530.8 eV peak is attributed to the lattice oxygen, while 531.7 eV peak is attributed to —OH or water molecule adsorbed on the surface of the 6CdSe/1rGO/3BN‐WO_3_ composites. The broad XPS peaks of N1s in Figure 3C are located at binding energy of ≈400.1 and ≈406.2 eV, and are attributed to the penta‐valent nitrogen. Our results are in good agreement with the previous reports about N‐doped TiO_2_.^[^ ^54^ ^]^ Figure 3D exhibits the B1s spectrum and the peak at 193.6 eV is clearly above the statistical noisy level, providing solid evidence for the existence of B^3+^ in WO_3_ crystal, consistent with the previous reported peak position for B 1s.^[^ ^55^ ^]^ The C1s spectrum is deconvoluted into two peaks at 284.9 and 285.7 eV,^[^ ^60^ ^]^ which are attributed to C—C, and C—O in Figure S4B, Supporting Information. The XPS spectrum of Cd is shown in Figure 3E with the appropriate binding energies at 405.4 and 412.3 eV, which are related to the binding energy of Cd 3d_5/2_ and Cd 3d_3/2_, respectively. The XPS spectrum of a Se 3d is a very wide peak and centered at 52.3 eV as shown in Figure 3F. The low intensities of N1s, B1s, Cd3d, and Se3d are attributed to their small amounts in the nanocomposite. Based on XPS, XRD, UV–visible absorbance spectra TEM/HRTEM, and elementary mapping, it is concluded that the novel photocatalyst 6CdSe/1rGO/3BN‐WO_3_ has been successfully synthesized.

### 3.3 Photocatalytic Activities

**Figure 4:**
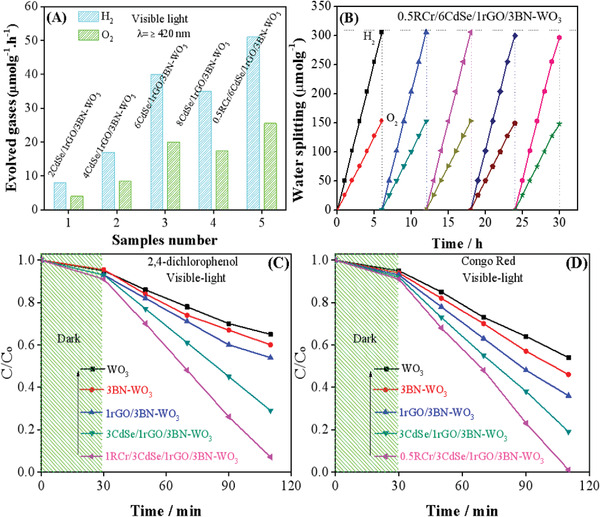
Visible light photocatalytic activities for overall water splitting with 2CdSe/1rGO/3BN‐WO_3_, 4CdSe/1rGO/3BN‐WO_3_, 6CdSe/1rGO/3BN‐WO_3_, 8CdSe/rGO/3BN‐WO_3_, and 0.5RCr/6CdSe/1rGO/3BN‐WO_3_ (A), stability and recyclability test of the sample‐optimized 0.5RCr/6CdSe/1rGO/3BN‐WO_3_ (B), visible‐light photocatalytic degradation activities for dichlorophenol (C), and Congo Red of WO_3_, 3BN‐WO_3_, 1rGO/3BN‐WO_3_, 6CdSe/1rGO/3BN‐WO_3_, and 0.5RCr/6CdSe/1rGO/3BN‐WO_3_ (D).

The overall water splitting photocatalytic activities of WO_3_, *x*BN‐WO_3_, and yCdSe/1rGO/3BN‐WO_3_ were measured with a directly connected GC‐system. It is clear from Figure S5, Supporting Information, that pure WO_3_, *x*BN‐WO_3,_ (where *x* represents different concentration) 1rGO/3BN‐WO_3_, and 1CdSe/1rGO/3BN‐WO_3_ are not suitable for water‐splitting activity, due to their unsuitable conduction band (CB near 0.26 eV) position and negligible visible‐light (*λ* ≥ 420 nm) absorption. However, after coupling CdSe QDs, the yCdSe/1rGO/3BN‐WO_3_ samples demonstrated much better overall water splitting activities as given in **Figure** **4**A and the 6CdSe/1rGO/3BN‐WO_3_ sample exhibited very high activity (40/20 (H_2_/O_2_) µmol h^−1^ g^−1^).

In order to further enhance the photocatalytic activities, the optimized sample was modified with 0.5RCr as co‐catalyst by a photo‐deposition process. The sample 0.5RCr/6CdSe/1rGO/3BN‐WO_3_ showed surprisingly high overall water splitting activities (i.e., 51/25.5 (H_2_/O_2_) µmol h^−1^ g^−1^). Moreover, we also measured the stability and recyclability test for the sample‐optimized. Obviously, there is no any reasonable reduction in the photocatalytic activity after a 5‐cycle (30 h) run as demonstrated in Figure 4B. Thus, the newly designed nanocomposites are highly stable and efficiently active for overall water splitting.

In addition, we further studied the photocatalytic oxidation properties of the nanocomposites through controlled 2,4‐dichlorophenol and Congo Red degradation activities. Since, adsorption of the pollutants on the photocatalyst surface can play a crucial role in the degradation of toxic materials, therefore, we carried out experiments in the dark for 30 min to complete adsorption–desorption and then irradiated the samples under visible light. As a result, the 0.5RCr/6CdSe/1rGO/3BN‐WO_3_ nanocomposite showed 91% photodegradation activities for 2,4‐dichlorophenol degradation in 1.5 h as shown Figure 4C. It is evidently observed that the 2,4‐dichlorophenol degradation results are supporting the overall water splitting activities, suggesting that the sample‐optimized 0.5RCr/6CdSe/1rGO/3BN‐WO_3_ nanocomposite exhibits high photo‐degradation activities.

Similarly, the sample‐optimized showed very attractive photoactivity for the Congo Red degradation and ≈99% Congo Red was degraded in 1.5 h under visible‐light irradiation as mentioned in Figure 4D. These results show that the as synthesized nanocomposite 0.5RCr/6CdSe/1rGO/3BN‐WO_3_ has great redox performance for the overall water splitting to evolve H_2_ and O_2_, and for the pollutants degradation. Thus, it is confirmed that the band gap engineering and extended visible light absorption are responsible for the improved photocatalytic activities.

### 3.4 Charge Separation and Mechanism Discussion

The exploration and investigation of photo‐generated charge carrier properties of nano‐sized photo‐semiconductors with negligible band bending is an exciting task. Photoluminescence (PL) spectroscopy is a versatile and unique technique used to explore photochemical properties of energetic sites on the surface of photocatalysts. From the PL spectra, we can also get perception about the surface defects and oxygen vacancies, and charge transfer and charge carrier trapping efficiency. Figure S6A,B, Supporting Information, shows the PL spectra of WO_3_, *x*BN‐WO_3_, and yCdSe/3BN‐WO_3_ samples under an excitation wavelength of 420 nm. It is observed that after (B,N) co‐doping and coupling with CdSe QDs, the intensities of PL spectra are further decreased. Interestingly, the PL intensity is efficiently decreased after the photodeposition of a co‐catalyst RCr. Thus, 0.5RCr/6CdSe/1rGO/3BN‐WO_3_ nanocomposite demonstrates low charge recombination and an efficiently high charge separation, which is consistent with photocatalytic activities.

**Figure 5:**
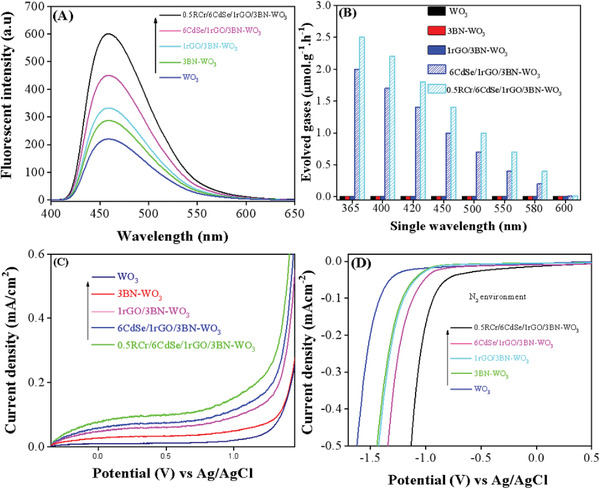
•OH radical amount‐related fluorescence spectra (A), single wavelength activities (B), photoelectrochemical current density (C), and electrochemical reduction curves of WO_3_, 3BN‐WO_3_, 1rGO/3BN‐WO_3_, 6CdSe/1rGO/3BN‐WO_3_, and 0.5RCr/6CdSe/1rGO/3BN‐WO_3_ (D). Electrochemical performance was measured in a 0.5 m NaClO_4_ solution and Hg/Hg_2_Cl_2_ (saturated KCl) electrode was used as the reference electrode.

To extend the charge separation techniques, coumarin fluorescence spectroscopy was utilized to investigate the amount of hydroxyl radicals (•OH) by measuring the spectra of luminescent 7‐hydroxycoumarin formed by the chemical reaction between coumarin and •OH. It has been observed and accepted that the signal intensity strength is proportional to the •OH radicals produced and charge separation. As shown in the **Figure** **5**A, the amount of produced •OH by 3BN‐WO_3_ and 1rGO/3BN‐WO_3_ is greater than that of pure WO_3_, and its signal intensity is substantially increased after the introduction of CdSe QDs. The produced •OH amount is specifically larger for 6CdSe/1rGO/3BN‐WO_3_. Furthermore, the amount of •OH is greatly enhanced by adding a co‐catalyst such as 0.5RCr/6CdSe/1rGO/3BN‐WO_3_ due to the interfacial charge transfer through the Z‐scheme mechanism.

To confirm the extended visible‐light activity of 0.5RCr/6CdSe/1rGO/3BN‐WO_3_, we performed single wavelength photocatalytic water splitting activities as given in Figure 5B. According to the results, pure WO_3_ has no activity, because the CB and VB level are unsuitable and therefore it does not show any photocatalytic activity. However, after co‐doping with BN and coupling CdSe QDs, the visible light response is increased up to 580 nm. It is noteworthy that the newly designed nanocomposite 0.5RCr/6CdSe/1rGO/3BN‐WO_3_ exhibits an excellent overall water splitting activity due to the absorption of a wide range of the solar spectrum.

Photoelectrochemical (PEC) linear sweep voltammetry (LSV) measurements were performed under visible light irradiation in a 0.5 m Na_2_SO_4_ electrolyte solution and the results are depicted in Figure 5C. As expected, WO_3_ exhibits a rather weak photocurrent density compared to 3BN‐WO_3_ and 1rGO/3BN‐WO_3_. However, a more intense photocurrent density is observed for 6CdSe/1rGO/3BN‐WO_3_ and the photocurrent density signal of 0.5RCr/6CdSe/1rGO/3BN‐WO_3_ electrode is much larger than that of the other samples which further supports the idea of a band gap matched mechanism. Therefore, it could be observed that Z‐scheme interfacial matching and band gap bending via 3BN‐WO_3_ and 6CdSe QDs coupling plays an extremely significant role in enhancing charge separation of the photogenerated species.

Furthermore, the electrochemical reduction curves were employed to verify the catalytic function in nitrogen gas‐bubbled systems and demonstrate the mechanism of H_2_ evolution in the N_2_ environment as given in Figure 5D. It is more effective for electrochemical H_2_ evolution on 6CdSe/1rGO/3BN‐WO_3_ in comparison with pristine WO_3_. It is further endorsed by the interfacial Z‐scheme charge transfer between 3BN‐WO_3_ and 6CdSe QDs with a cocatalyst 0.5RCr. Since the sample‐optimized 0.5RCr/6CdSe/1rGO/3BN‐WO_3_ curve is shifted to a low onset in the N_2_ environment, the phenomenon is thermodynamically favorable for overall water splitting.

In addition, to deeply investigate charge transfer and separation mechanism, electrochemical impedance spectra (EIS) were obtained as shown in Figure S7, Supporting Information. A slight/detectable decrease is clear in the capacitive radius plots for 3BN‐WO_3_, 1rGO/3BN‐WO_3_ 6CdSe/1rGO/3BN‐WO_3_, and 0.5RCr/6CdSe/1rGO/3BN‐WO_3_ in comparison with pristine WO_3_. In specific, 0.5RCr/6CdSe/1rGO/3BN‐WO_3_ demonstrates the smallest arc radius, which represents the most crucial separation of photoinduced charges and carrier transfer from 3BN‐WO_3_ to CdSe QDs in the nanocomposite. Based on the interpretation of the PL spectra, •OH radical amount related spectra, single wavelength activities, photoelectrochemical (PEC) linear sweep voltammetry (LSV), electrochemical reduction curves, and electrochemical impedance spectra (EIS), it is concluded that the novel nanocomposites have efficiently high charge separation and transfer properties, which is consistent with the enhanced photocatalytic activities.

**Figure 6:**
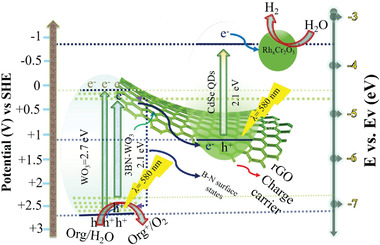
Proposed charge separation mechanism of the Z‐scheme system for 0.5RCr/6CdSe/1rGO/3BN‐WO_3_.

To well elucidate the underlying charge separation mechanism and improved visible light activities of the fabricated samples, a schematic mechanism is proposed, as shown in **Figure** **6**. Pristine WO_3_ has a band gap of ≈2.7 eV, corresponding to the light absorption threshold of 460 nm, which is certainly not good for visible light absorption. As we have discussed in the introduction, the CB position of pristine WO_3_ is below the water reduction level, leading to hydrogen evolution reaction (HER) to be impossible to photoexcited electrons, although the VB position of pristine WO_3_ is deep enough thus still enabling water oxidation through oxygen evolution reaction (OER) by photoexcited holes. The purpose of (B,N) co‐doping is to effectively reduce the band gap from 2.7 to 2.1 eV to extend the light absorption into the visible light range of 580 nm (≈2.1 eV). On the other hand, the CdSe QD shows a favorable position for HER due to its suitable CB position,^[^ ^7^ ^]^ thus with the help of the co‐catalyst (0.5%Rh*_x_* Cr_2_O_3_) the HER can be very effective. Unfortunately, the VB position of CdSe QD is too shallow for water oxidation, thus it is not a good OER catalyst.^[^ ^7^ ^]^ Therefore, the core idea of this project is to bring together (B,N) co‐doped WO_3_ and CdSe QD to construct a Z‐scheme system, in which the deep‐level photoexcited holes in (B,N) co‐doped WO_3_ helps OER and high‐energy photoexcited electrons in CdSe QD with co‐catalyst facilitates HER, leading to excellent efficiency for overall water splitting. Here we reiterate that the role of graphene is very critical because it helps to transport the low‐energy photoexcited electrons to be recombined with the shallow‐level photoexcited holes in CdSe, thus helping to increase the overall efficiency of charge separation for the Z‐scheme system of a (B,N) co‐doped WO_3_/CdSe QD‐RCr nanocomposite. To further confirm this idea, we show the Tauc‐plot and XPS valence band spectra (Figures S8A and S8B, Supporting Information) for the obtained 6CdSe/1rGO/3BN‐WO_3_ sample. It is clear that the VB position is ≈2.1 eV, considering the band gap of 2.3 eV, leading to the CB of 3BN‐WO3 equals 2.3 − 2.1 = 0.2 eV in vacuum level, which is 0.2 eV below the HER potential, which is consistent with our discussion in Figure 6. As a result, for the obtained 0.5RCr/6CdSe/1rGO/3BN‐WO_3_ nanocomposite, it exhibits the highest charge separation and the best photocatalytic performance, up to 580 nm of visible‐light irradiation. The novelty of this newly developed system is to excite both components of the Z‐scheme system at the same energy photon. In this case, both materials (B,N)‐WO_3_ and CdSe QDs have similar bang gaps (2.1 eV) and can easily be excited with the same energy photon, leading to excellent visible‐light overall water splitting efficiency.

## 4 Conclusions

In summary, a novel Z‐scheme system based on a band gap adjusted visible‐light responsive 0.5RCr/6CdSe/1rGO/3BN‐WO_3_ nanocomposite has been successfully constructed via hydrothermal method. Compared with the pristine WO_3_, the 0.5RCr/6CdSe/1rGO/3BN‐WO_3_ nanocomposite exhibited enhanced photocatalytic activities for the overall water splitting to evolve H_2_ and O_2_ and photodegradation for 2,4‐dichlorophenol and Congo Red pollutants. The improved photocatalytic activities of photocatalysts are ascribed to the extended visible‐light absorption due to (B,N) co‐doping to create surface resonant states near the CB and VB of WO_3_, and further promote the carrier's separation by coupling CdSe QDs and photo‐depositing RCr co‐catalyst. The Z‐scheme based mechanism followed by 6CdSe/1rGO/3BN‐WO_3_ nanocomposite is responsible for the high charge separation, and displays auspicious applications in photocatalysis for environmental remediation and energy conversion. This work demonstrates a promising approach to synthesize nanophotocatalysts based on WO_3_ for visible‐light driven solar energy application.

## 5 Experimentation Section

### Materials

Tungsten hexachloride WCl_6_ (99%), deduced graphene oxide (rGO), absolute ethanol (C_2_H_5_OH), methanol (CH_3_OH), and some gases (Ar, CO, N_2_, O_2_ [99.99%]) were purchased from Aladdin Chemistry Corporation Ltd, and EMD Millipore (Shanghai, China), respectively. Tetranitratoborate [(B(NO_3_)_4_]^−^ (99%) and Se were bought from Sinopharm Chemical Reagent Co. Ltd. Cadmium oxide, CdO (99.998%), octadecylphosphonic acid, C_18_H_39_O_3_P (ODPA 99%), and trioctylphosphine oxide, C_24_H_51_OP (TOPO 99%), were received from Sigma‐Aldrich. All the regents involved in the experiment were of analytical grade and directly utilized without further purification.

### Synthesis WO_3_ and BN‐WO_3_

The typical synthesis procedure as given: 3 g WCl_6_ were briskly stirred in 180 mL of absolute ethanol for 30 min. With continues agitation, the solution first became translucent followed by pale yellow coloration. The pale‐yellow solution was subsequently processed in 100 mL Teflon‐lined autoclaves and kept in an oven at 180 °C for 24‐h for the hydrothermal preparation of WO_3_ nanoparticles. The obtained sediments were washed numerous times with deionized water and absolute C_2_H_5_OH in order to completely remove all the residues followed by calcination at 600 °C for 2‐h in air to extract water molecules and get WO_3_ nanoparticles. For the preparation of BN co‐doped pristine WO_3_ nanoparticles, the same procedure was followed except for the addition of different percentages of tetranitratoborate [(B(NO_3_)_4_]^−^ (99%) as a source of BN and the obtained products were denoted by *x*BN‐WO_3_ where *x* shows the different weight percent of tetranitratoborate [(B(NO_3_)_4_]^−^.

### Modification and Coupling

To fabricate rGO (1%) modified and CdSe QDs (2‐8%) coupled photocatalysts, the typical procedure followed was as follows: 0.5 g 3BN‐WO_3_ were stirred in 40/40 mL of ethanol/distilled water mixture in a 150 mL glass beaker followed by the addition of 1% rGO and the vigorous stirring was continued for the next 30 min to form a homogeneous mixture. Subsequently, desirable quantities of CdSe QDs (0.5 mg mL^−1^), (2, 4, 6, and 8 mL) were added and the ultrasonic treatment was continued for 3 h at ambient temperature. Eventually, each mixture was held at 80 °C for ≈3 h to dehydrate. The final product was exposed to the heat treatment process at 300 °C moderately (1 °C min^−1^ in air) for 2 h and the samples were denoted by *y*CdSe/1rGO/3BN‐WO_3_ where *y* represents the weight percent of CdSe QDs in the nanocomposite.

### Photodeposition of Rh*_x_* Cr_2_O_3_

In an attempt to enhance the photocatalytic activity of the optimized (6CdSe/1rGO/3BN‐WO_3_) sample, RhCl_3_ and Cr(NO_3_)_3_ as cocatalysts were pursued through the experiment. Before starting photodeposition, a mixture of 100 mL methanol and 100 mL deionized water was enriched by Ar gas to eliminate oxygen from the solution. Thereafter, a 0.5 g photocatalyst was mixed with 40 mL of the above‐mentioned solution. The photodeposition of 0.5RCr on the 6CdSe/1rGO/3BN‐WO_3_ sample was achieved by dropping technique with continuous vigorous stirring under a Xe‐lamp‐light irradiation for 1 h. After centrifugation and washing it several times for the removal of the residual material, the obtained sample was dried at 80 °C overnight.

### Material Characterization

XRD patterns were analyzed at a scanning speed of 12° min^−1^ in the X‐ray diffractometer with Cu‐K*α*1 emission (*λ* = 1.5406 Å). The XPS (VG Scientific, Model ESCALAB250) was used to analyze the surface configuration and elemental oxidation states of the as prepared samples. TEM (JEM‐2100 Plus) was used to investigate the structure morphology of the materials. The HR‐TEM, EDS, and SAED of CdSe QDs coupled rGO loaded WO_3_ were obtained using a Cs‐corrected Model JEOL JEM‐2200FS microscope operated at 200 kV. The surface morphology of nanocomposite was examined with the scanning electron microscope (SEM) (EM8000, KYKY, China) equipped with energy‐dispersive X‐ray spectroscopy (EDX) (Bruker, Germany). The UV–Vis absorption of the nanocomposites was detected with Shimadzu UV‐2550 Spectrophotometer, using white BaSO_4_ powder as a reference. The photoluminescence (PL) spectra were received from PE‐LS‐55 Spectrofluorophotometer (excitation wavelength *λ* = 420 nm).

### Overall Water Splitting Evaluation

The overall water splitting activity was carried out inside a transparent cylindrical quartz cell (250 mL m^−3^), which was associated to a gas circulation system and TCD (Tech, GC‐7900, nitrogen carrier) to measure the evolved gases. For photocatalytic activity measurement, 50 mg samples were disseminated in 100 mL distilled water and exposed to light irradiation under a 300 W Xe‐lamp (cut‐off below of 420 nm). The amount of evolved gases was measured at an interval of a 1 h duration.^[^ ^56^, ^57^ ^]^

### Measurement of Hydroxyl Radical (•OH) Amount

For the measurement of •OH amount, a 50 mg sample was dispersed in 20 mL of a 5 mg L^−1^ coumarin aqueous solution in a quartz reactor. The chemical reaction was monitored under a strong magnetic stirring for 1 h under light irradiation with a Xe‐lamp (300 W) having a cut‐off filter (*λ* ≥ 420 nm) and the Xe‐lamp was placed at about a 12 cm height from the reactor chamber. Ultimately, a portion of the solution under investigation was separated by centrifugation and transferred to a Pyrex glass cell in order to investigate the fluorescence of 7‐hydroxycoumarin at a 420 nm excitation wavelength.

### Evaluation of Pollutants Degradation

The representative procedure for 2,4‐dichlorophenol photocatalytic degradation was given as: 50 mg of the catalyst was dispersed in 80 mL of 2,4‐dichlorophenol solution (10 mg L^−1^). Thereafter, the mixture was stirred in a dark environment for 30 min to ensure adsorption–desorption saturation. Before centrifugation, the mixture was exposed to light irradiation under visible light and samples were collected after an appropriate time interval. Finally, the 2,4‐dichlorophenol concentration was measured by the UV‐spectrophotometer with an absorption peak at 285 nm. A similar procedure was used for the Congo Red degradation.^[^ ^58^, ^59^ ^]^

## Conflict of Interest

The authors declare no conflict of interest.

## Supporting information

Supporting Information

## Data Availability

Research data are not shared.
