# Supplementary material for: Exceptional Photocatalytic Activities of rGO Modified (B,N) Co‐Doped WO3, Coupled with CdSe QDs for One Photon Z‐Scheme System: A Joint Experimental and DFT Study
Source: Adv Sci (Weinh). 2021 Dec 3;9(2):2102530. doi: 10.1002/advs.202102530 (PMC8805570; doi:10.1002/advs.202102530)
Supplement: Supplementary file 1 — Supporting Information [file ADVS-9-2102530-s001.pdf]

© 2021 Wiley-VCH GmbH

ADVANCED SCIENCE Open Access

Supporting Information

for *Adv. Sci.*, DOI: 10.1002/advs.202102530

Exceptional Photocatalytic Activities of rGO Modified (B,N) Co-doped WO3, Coupled with CdSe QDs for one Photon Z-Scheme System: A Joint Experimental and DFT Study

Fazal Raziq[1,2a], Amil Aligayev[2a], Huahai Shen[3], Sharafat Ali[1,2], Rahim Shah[2], Sajjad Ali[4], Syedul H. Bakhtiar[5], Asad Ali[6], Naghat Zarshad[6], Amir Zada[6], Xiang Xia[2], Xiaotao Zu[2], Muslim Khan[7], Xiaoqiang Wu[8], Qingquan Kong[8], Chunming Liu[2], Liang Qiao[1,2]**

Supporting in formation

Exceptional Photocatalytic Activities of rGO Modified (B,N) Co-doped WO_3, Coupled with CdSe QDs for one Photon Z-Scheme System: A Joint Experimental and DFT Study

Fazal Raziq[1,2a], Amil Aligayev[2a], Huahai Shen[3], Sharafat Ali[1,2], Rahim Shah[2], Sajjad Ali[4], Syedul H. Bakhtiar[5], Asad Ali[6], Naghat Zarshad[6], Amir Zada[6], Xiang Xia[2], Xiaotao Zu[2], Muslim Khan[7], Xiaoqiang Wu[8], Qingquan Kong[8], Chunming Liu[2]*, Liang Qiao[1,2]*

[1]*Yangtze Delta Region Institute (Huzhou)，University of Electronic Science and Technology of China, Huzhou 313001, P. R. China.*

[2]*School of Physics, University of Electronic Science and Technology of China, Chengdu, 610054, P. R. China.*

[3]*Institute of Nuclear Physics and Chemistry, Chinese Academy of Engineering Physics, Mianyang, 621900, P. R. China.*

[4]*Department of Physics, Southern University of Science and Technology, 518055 Shenzhen, China.*

[5]*The State Key Laboratory of Advanced Technology for Materials Synthesis and Processing, Wuhan University of Technology, Wuhan, 430070, P. R. China.*

[6]*Department of Chemistry, Abdul Wali Khan University Mardan, KPK, Pakistan.*

[7]*Department of Chemistry, Kohat University of Science and Technology, Kohat, KPK, Pakistan.*

[8]*School of Mechanical Engineering, Chengdu University, Chengdu, 610106, China*

# Fazal Raziq and Amil Aligayev contribute equally.

*Corresponding author email: cmliu@uestc.edu.cn, liang.qiao@uestc.edu.cn

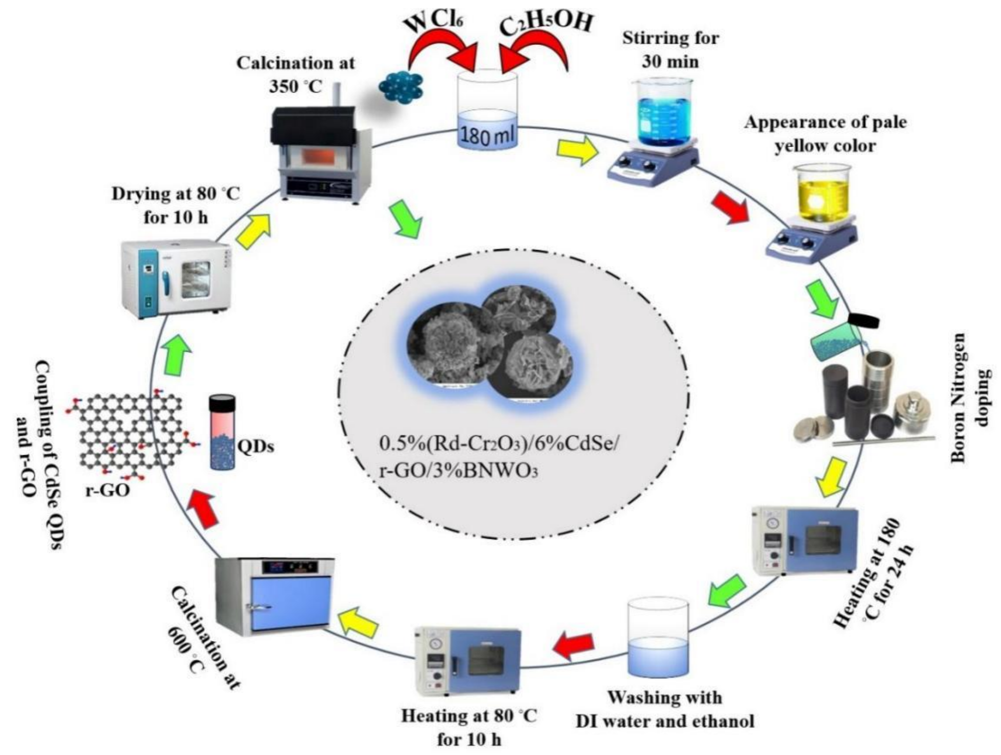

SI-Scheme 1: The schematic presentation of experimental procedure.

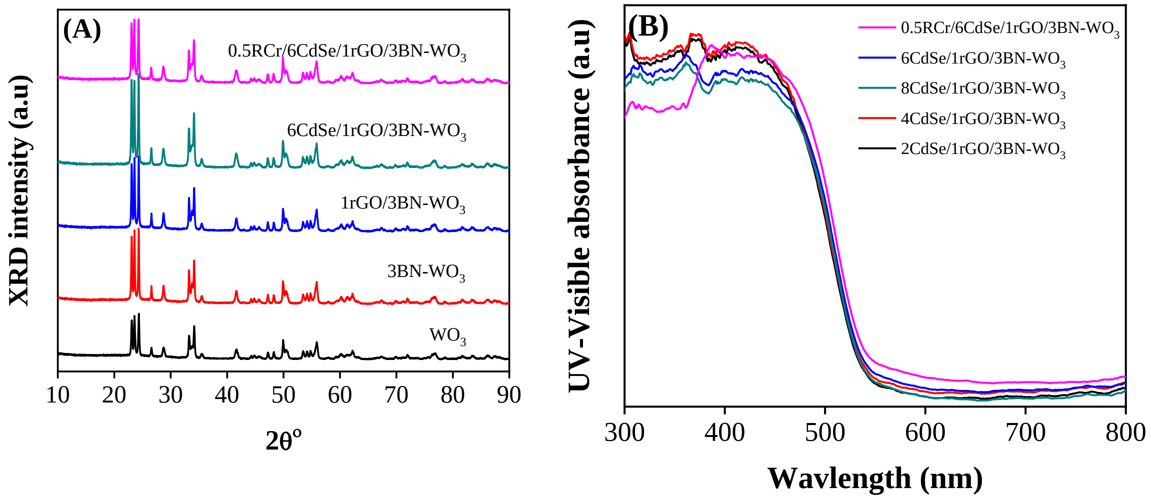

SI-Figure 1: XRD patterns of WO_3, 3BN-WO_3, 1rGO/3BN-WO_3, 6CdSe/1rGO/3BN-WO_3 and 0.5RCr/6CdSe/1rGO/3BN-WO_3 (A), UV-Visible absorbance spectra of

2CdSe/1rGO/3BN-WO_3, 4CdSe/1rGO/3BN-WO_3, 6CdSe/1rGO/3BN-WO_3 and 0.5RCr/6CdSe/1rGO/3BN-WO_3 (B).

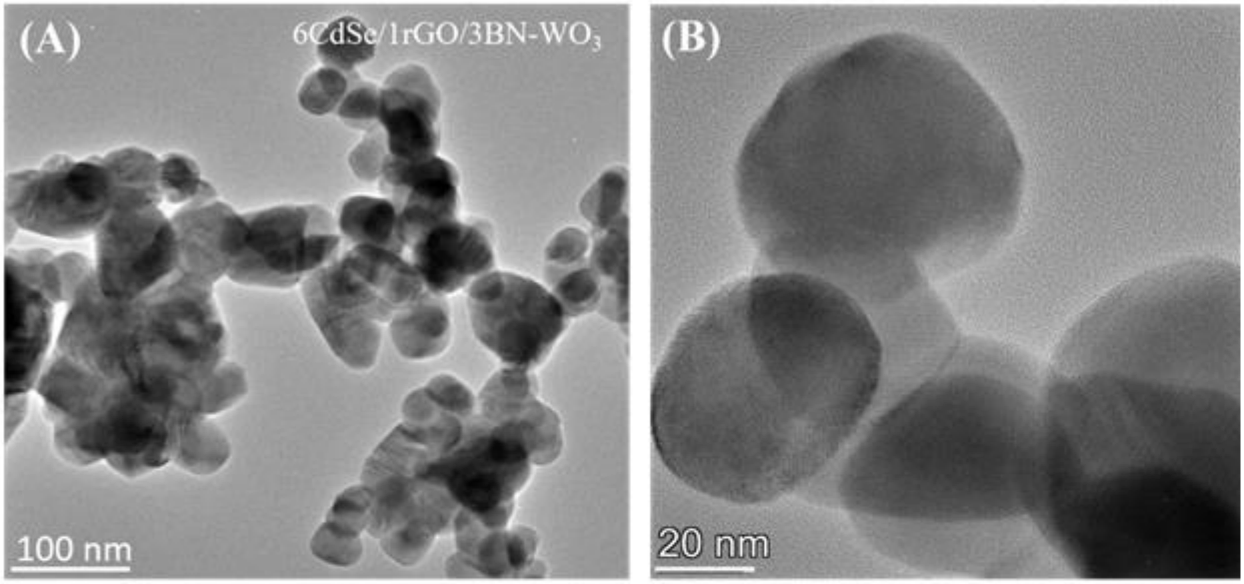

SI-Figure 2: TEM images of 6CdSe/1rGO/3BN-WO_3

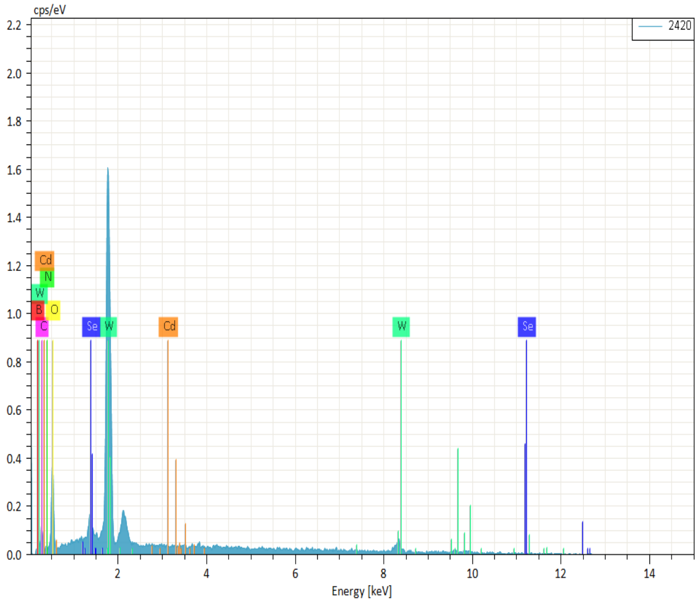

SI-Figure 3: EDX of 6CdSe/1rGO/3BN-WO_3

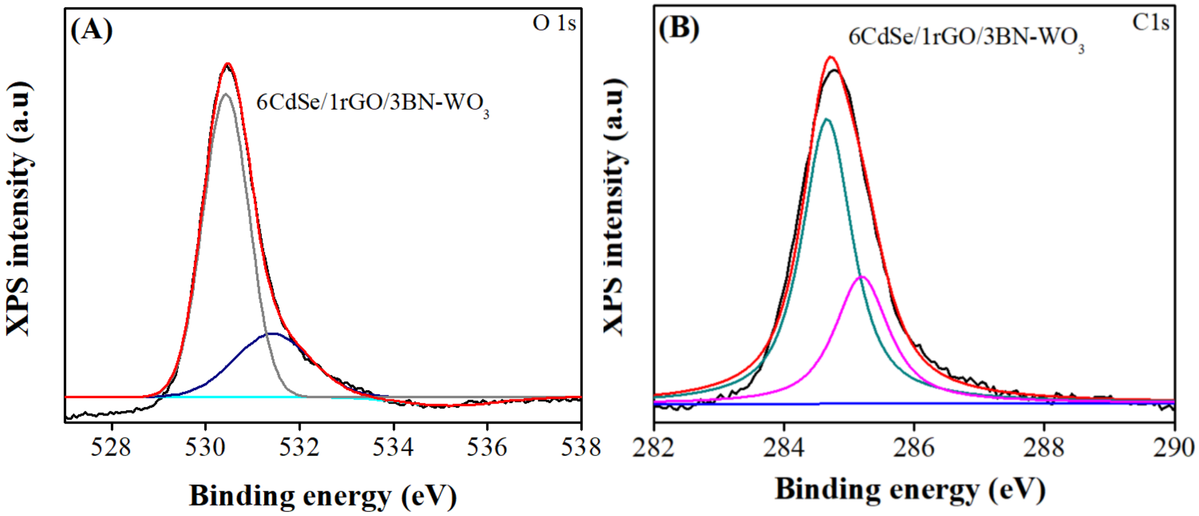

SI-Figure 4: The XPS spectrum (A) O1s and (B) C1s of 6CdSe/1rGO/3BN-WO_3

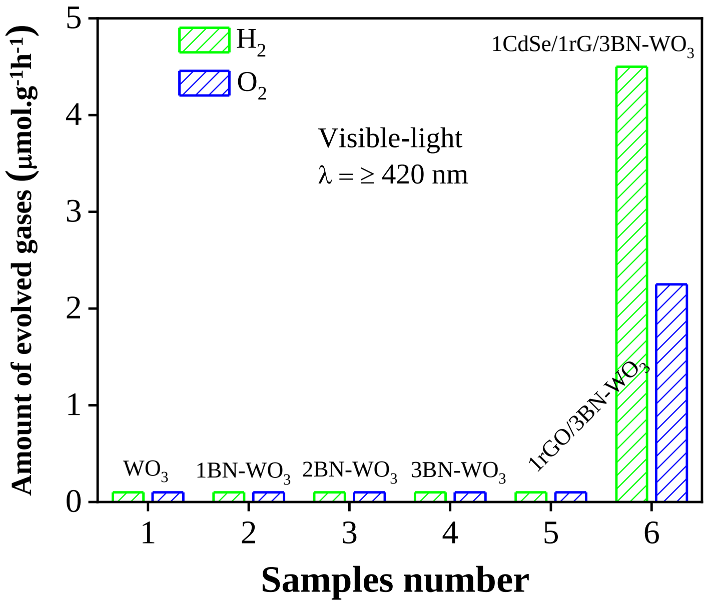

SI-Figure 5: Visible light photocatalytic activities of overall water splitting for WO_3, 1BN-WO_3, 2BN-WO_3, 3BN-WO_3, 1rGO/3BN-WO_3 and 1CdSe/1rGO/3BN-WO_3.

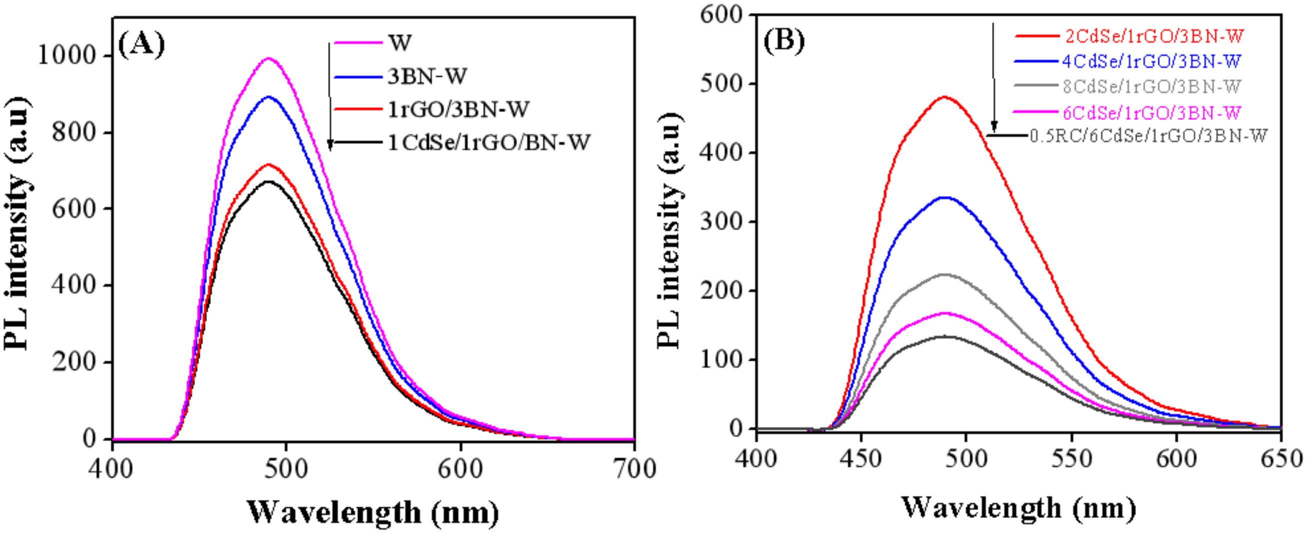

SI-Figure 6: Photoluminescence spectra (A) WO_3, 3BN-WO_3, 1rGO/3BN-WO_3 and 1CdSe/1rGO/3BN-WO_3 and (B) of 2CdSe/1rGO/3BN-WO_3, 4CdSe/1rGO/3BN-WO_3, 6CdSe/1rGO/3BN-WO_3, 8CdSe/1rGO/3BN-WO_3 and 0.5RCr/6CdSe/1rGO/3BN-WO_3. The photoluminescence spectra demonstrated that after BN doping and rGO modification the rate of charge recombination decreased. Further the charge separation and transfer improved with CdSe QDs coupling and 0.5RCr photodeposition modification. The sample-optimized 0.5RCr/6CdSe/1rGO/3BN-WO_3 showing lowest peak intensity. Thus 0.5RCr/6CdSe/1rGO/3BN-WO_3 have lower carriers' recombination and high charge separation. (WO_3=W)

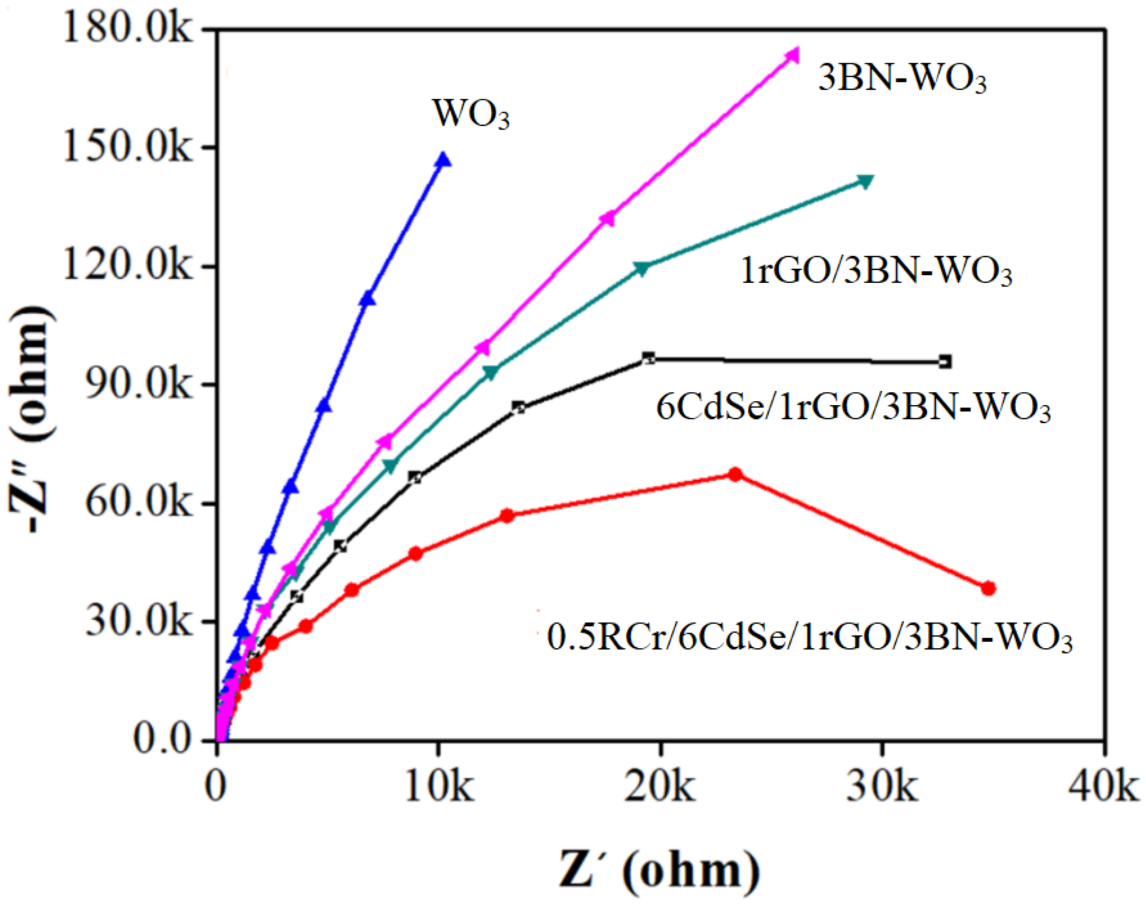

SI-Figure 7: Electrochemical impedance spectra Nyquist plots of WO_3, 3BN-WO_3, 1rGO/3BN-WO_3, 6CdSe/1rGO/3BN-WO_3 and 0.5RCr/6CdSe/1rGO/3BN-WO_3 in visible-light at the applied potential of -0.4 V vs Ag/AgCl in 0.5 M Na_2SO_4 solution.

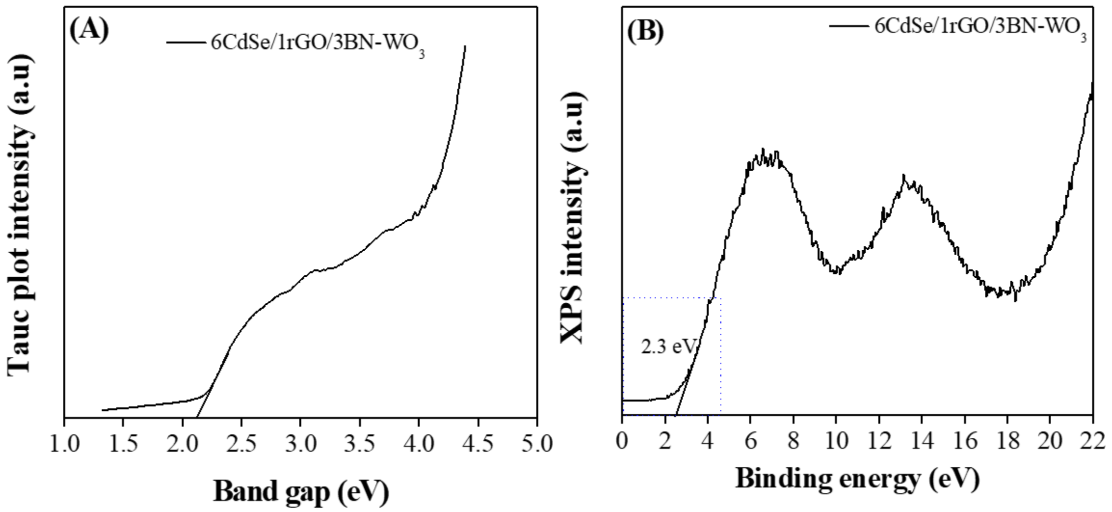

SI-Figure 8: Tauc plat (A) for bandgap calculation and XPS valence spectra of 6CdSe/3BN-WO_3
